# CALLING OUT AGEISM: HOW INTERSECTING IDENTITIES AND CONFRONTATION TONE AFFECT PERCEPTIONS OF OLDER CONFRONTERS

**DOI:** 10.1093/geroni/igae098.0016

**Published:** 2024-12-31

**Authors:** Hannah Gans, Alison Chasteen, Michelle Horhota

**Affiliations:** University of Toronto, Toronto, Ontario, Canada; University of Toronto, Toronto, Ontario, Canada; Furman University, Greenville, South Carolina, United States

## Abstract

The few studies that have examined confrontations of ageism have shown that older adults are viewed differently after challenging the behavior. What remains unclear is how older people’s intersecting identities, as well as their confrontation tone, might influence reactions to their countering ageism. To address this question, we surveyed 500 young and older adults regarding their perceptions of warmth and competence towards older adult confronters who varied by race (Black, White) and gender (man, woman). Participants evaluated the older individuals both before and after they either moderately or strongly confronted an ageist backhanded compliment (“You don’t look that old”). The older confronter’s race and their response to the ageist comment moderated how they were perceived by participants. Specifically, both young and older adult participants rated older Black confronters as warmer when they used a moderate compared to a strong confrontation tone but did not make that distinction for White confronters. However, when the tone of the confrontation was not considered, young, and older participants generally differed in their perceptions of confronters. Older adult participants rated White confronters lower on warmth and competence after confronting compared to Black confronters. This pattern was not observed among young adult participants, underscoring the significance of intersectional identities in shaping perceptions among older adult perceivers. This study shows that older adult confronters’ intersecting identities influence how they are perceived when they challenge ageist comments. This suggests that customized confrontation techniques may be needed for older adults whose gender and racial identities vary.

